# Targeted therapies in biliary tract cancer—when precision becomes imprecise

**DOI:** 10.1016/j.esmogo.2024.100085

**Published:** 2024-08-13

**Authors:** C.J. O’Rourke, J.V. Schou, J.B. Andersen, D. Høgdall

**Affiliations:** 1 Biotech Research and Innovation Centre (BRIC), Department of Health and Medical Sciences, University of Copenhagen, Copenhagen; 2 Department of Oncology, Herlev and Gentofte Hospital, Herlev, Copenhagen University Hospital, Copenhagen, Denmark

**Keywords:** biliary tract cancer, genomics, predictive biomarkers, primary resistance, targeted therapies

## Abstract

Advanced biliary tract cancers (BTCs) have gained notoriety among gastrointestinal tumours for their comparatively high incidence of actionable alterations and their compelling benefit from targeted therapies matched to these alterations. Such successes are exemplified by BTC-specific approvals of fibroblast growth factor receptor (FGFR) inhibitors for tumours with *FGFR2* rearrangements, as well as mutant isocitrate dehydrogenase 1 inhibitors. Nevertheless, there is a clear absence of therapeutic benefit in a subset of patients despite their tumours fulfilling the current molecular criteria for treatment with these drugs. This results in inefficient management of patients with otherwise bleak prognosis, as well as considerable financial burden. Even among responders, the duration of response is limited, a clinical observation that could be considered unusual as these inhibitors typically target driver genes hypothesised to be responsible for tumour formation. However, BTCs exhibit oncogenic addiction to signalling networks rather than individual genes, and by extension, therapeutic response is dependent on these signalling networks rather than simply the status of the specific target gene. Primary resistance is mediated by co-occurring genetic (DNA) and non-genetic (transcriptional, translational, post-translational) alterations in members of signalling networks that are upstream, downstream, or in parallel pathways to the target alteration. Refining the molecular criteria to select patients is a necessary next step, by incorporating co-occurrence of resistance biomarkers as individual parameters or into predictors of treatment benefit. Characterising the molecular bases of resistance to targeted therapies will fuel next-generation combination treatments, maximising the catchment of responders and enhancing the duration of response.

## Highlights

• Targeted therapies revolutionized biliary tract cancer management but some tumours harbouring the target do not respond.
• Primary resistance can arise due to co-occurring alterations affecting the target gene and its pathways.
• Refining molecular selection criteria to include resistance biomarkers can improve patient response rates and outcome.
• Understanding primary resistance mechanisms will provide biological rationale for the design of novel combination regimens.

## Introduction

Management of biliary tract cancer (BTC) has historically relied on systemic chemotherapy.^1^ Gemcitabine–cisplatin^2^ with or without checkpoint inhibitors^3^^,^^4^ is the current first-line standard of care for patients with recurrent, locally advanced, or metastatic disease. Such a universal approach up-front is challenged by the widespread heterogeneity of BTC, including diagnostic (cholangiocarcinoma, gallbladder carcinoma), anatomical (intrahepatic, perihilar, distal), histomorphological (large or small duct, growth pattern), and molecular (genetic, non-genetic) differences.^5^ BTC originally emerged into the spotlight as remarkably responsive to precision medicine approaches in basket trials,^6^ exhibiting high incidence of actionable alterations and therapeutic benefit when treated for these alterations compared to other cancers.^7^ It is now clear that biomarker-driven selection of BTC patients for targeted therapies improves overall survival compared to those without,^8^ cementing tumour molecular profiling as fundamental to the care of these patients.^9^

Following landmark BTC trials,^10^, ^11^, ^12^, ^13^, ^14^ as well as tumour-agnostic studies,^15^, ^16^, ^17^ multiple biomarker-guided targeted therapies are becoming available in the palliative setting after progression on chemotherapy. However, while current molecular selection of patients enriches for responders compared to the general population,^10^ subgroups of patients derive no therapeutic benefit from targeted therapies despite their tumours harbouring the target alteration (due to primary or innate resistance). Further, virtually all patients experience progression on targeted therapies within a relatively short timeframe (caused by secondary or acquired resistance). Therefore, our current molecular criteria for patient selection are suboptimal, requiring biology-driven refinement. This will improve individualised treatment planning, offset economic burden of prescribing high-cost drugs to patients inherently without treatment benefit, and guide the design of future combination strategies to enhance and prolong the duration of disease control.

In this article, we discuss the incidence of primary resistance to targeted therapies in BTC, including the molecular basis contributing to disease progression and future strategies to improve the use of targeted therapies.

## Targeted therapies in biliary tract cancer

**Figure 1:**
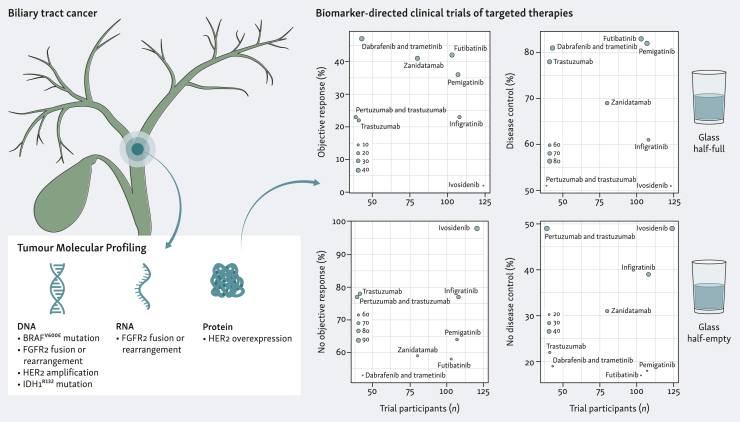
**Performance of molecularly matched targeted therapies in phase II/III trials for biliary tract cancer.** Molecular indication: *BRAF*^V600E^ mutation—dabrafenib and trametinib (NCT02034110); *FGFR2* fusion or rearrangement—futibatinib (NCT02052778), infigratinib (NCT02150967), pemigatinib (NCT02924376); *HER2* amplification or overexpression—pertuzumab and trastuzumab (NCT02091141), trastuzumab (NCT04482309), zanidatamab (NCT04466891); *IDH1*^R132^ mutation—ivosidenib (NCT02989857). DC, disease control; *FGFR2*, fibroblast growth factor receptor 2; *HER2*, human epidermal growth factor receptor 2; *IDH1*, isocitrate dehydrogenase 1; OR, objective response.

Landmark phase II and III trials have demonstrated the therapeutic benefit of molecularly matched targeted therapies in BTC after progression on chemotherapy^10^, ^11^, ^12^, ^13^, ^14^, ^15^, ^16^, ^17^ (Figure 1). Unquestionably, intrahepatic cholangiocarcinoma (iCCA) has dominated the targeted therapy trial space. Fibroblast growth factor receptor 2 (*FGFR2*) is perturbed by genomic rearrangements (including fusions) in ∼9% of iCCA.^18^ Among *FGFR2*-altered tumours, disease control was achieved in 82% (88/107) with pemigatinib,^10^ 83% (85/103) with futibatinib,^11^ and 75% (46/61) with infigratinib.^12^ Isocitrate dehydrogenase 1 (*IDH1*) is mutated in ∼14% of iCCA.^18^ Treatment with the mutant IDH1 inhibitor, ivosidenib, resulted in disease control for 51% (63/124) of patients and favourable overall survival compared to placebo.^13^ Human epidermal growth factor receptor 2 (*HER2*) is recurrently altered (amplification, mutation, overexpression) in approximately 25% of gallbladder cancer (GBC), 18% of extrahepatic cholangiocarcinoma (eCCA), and 4% of iCCA.^14^ Disease control occurred in 69% (55/80) of HER2-positive BTC patients with the bispecific antibody, zanidatamab.^14^ In basket trials, disease control occurred in BTC with additional targeted regimens: 81% (35/43) with dabrafenib plus trametinib targeting V600E mutations in proto-oncogene B-Raf (*BRAF*^V600E^)^15^; 78% (32/41) with trastuzumab targeting HER2-positive tumours^7^; 51% (20/39) with pertuzumab plus trastuzumab targeting HER2-positive/-altered tumours.^16^ While these studies have been or will likely be practice-changing, these targeted agents failed to achieve disease control in 29% (174/598) of patients despite their tumours testing positive for the actionable alteration. Further, objective response was not observed in 70% (418/598) of these patients. Granted these trials have increased the therapeutic arsenal for subgroups of patients, it is also evident that the current molecular inclusion criteria are suboptimal and the elusive factors responsible for undermining targeted therapy response should also be co-evaluated when making treatment decisions.

## Target status and primary resistance

Currently, precision approaches to targeted therapies require detection of a target genomic alteration (DNA) or its positive expression (RNA, protein) in tumour tissue. However, not all alterations in a target gene are created equally (for example, different breakpoints in fusions, different amino acid substitutions at a mutation hotspot), and such heterogeneity could undermine therapeutic benefit for patients.

Rearrangements of *FGFR2* function as drivers by constitutively activating pro-growth signalling independent of FGF ligands. This is accomplished by loss of the regulatory C-terminal domain of FGFR2 or by gain of domains from fusion partners that enhance FGFR2 dimerisation. Therapeutic response to FGFR inhibition requires intact tyrosine kinase domains and in-frame coding sequences after rearrangement. These molecular nuances are important, as depending on the molecular test used for molecular profiling, different levels of resolution are obtained.^19^ For example, fluorescence *in situ* hybridisation might be used in cases with limited tumour tissue but positive signal for *FGFR2* rearrangements does not necessarily indicate that the kinase domain is functional, or the alteration is in-frame, with implications for therapeutic response. It is also possible for multiple alterations to affect FGFR2 in a single sample, including co-occurrence of FGFR inhibitor (FGFRi) resistance-promoting point mutations^18^ that were previously only identified after progression on these drugs.^20^^,^^21^ Optimally, a sequencing-based approach spanning exons and introns of the entire *FGFR2* gene is needed to confirm potential on-target responsiveness.

Treatment benefit from inhibitors targeting oncogenic alterations is dependent on active transcription and translation of genetic variants into altered proteins, but this is not always the case. Approximately 26% (6/23) of cancer hotspot alterations discovered in BTC are not actively transcribed into messenger RNA (mRNA), with GBC having particular high incidence of such transcriptional silencing compared to other solid cancers.^22^ While some oncogenic alterations appear to always be transcribed (*KRAS*, *PIK3CA*, *TP53*), other actionable targets exhibit discordance between DNA variants and mRNA expression (*ALK*, *ERBB4*, *RET*).^22^ Further, out-of-frame fusions or introduction of premature stop codons can produce transcripts that are rapidly degraded.^19^ These data suggest that some biliary tumours are positive for a targetable DNA alteration but the altered variants are not subsequently expressed, resulting in primary resistance to the corresponding targeted therapy.

## Signalling network status and primary resistance

Oncogenic addiction is operative at the level of the signalling pathway and its interacting networks rather than the individual driver alteration. Therefore, alterations co-occurring within networks can influence primary response to targeted therapies in a subset of patients. For example, FGFR2 signalling relays extracellular growth stimuli to a series of intracellular downstream networks that are recurrently altered in BTC, including mitogen-activated protein kinase, phosphoinositide 3-kinase–alpha serine/threonine-protein kinase, and Janus kinase–signal transducer and activator of transcription pathways.

Targeted sequencing panels currently used for molecular profiling might already provide useful data that could be used to enhance patient selection for targeted therapies. For example, introducing G12D-mutant Kirsten rat sarcoma virus (*KRAS*^G12D^) is sufficient to blunt FGFRi response in *FGFR2* fusion-driven human^23^ and mouse models^23^^,^^24^ of iCCA; this indicates that these co-occurring alterations could be responsible for primary resistance in a subset of patients. Retrospective analysis of the FIGHT-202 trial was insufficient to determine this, as only one tumour harboured co-occurring *FGFR2* rearrangement and mutant *KRAS.*^7^ Larger studies have since confirmed that these alterations co-occur in ∼1.2% of iCCA,^18^ so careful investigation of their relative benefit from FGFRi is now needed. Similar FGFRi blunting effects have been reported for co-occurring *BRAF*^V600E^ mutations *in vitro*,^23^ so it appears likely that the dominant negative effects of co-occurring mutations may not be restricted to *KRAS* alone.

Beyond genomic co-alterations, epidermal growth factor (EGF) stimulation is also sufficient to blunt FGFRi response in preclinical models.^25^^,^^26^ Further mechanistic investigation clarified the EGF receptor (EGFR) network as an alternative signalling route (bypassing inhibited FGFR2 signalling) that activates mitogen-activated protein kinase kinase-MAPKK/extracellular signal-regulated kinase, which is responsible for mediating the oncogenic effects of FGFR2 alterations.^23^^,^^25^^,^^26^ By bypassing FGFR2 signalling via EGFR, downstream signalling (to which the tumour is addicted) becomes actively maintained in the presence of FGFRi, highlighting a primary resistance mechanism operative in a subset of iCCA. Whether the origins of such stimulation arise due to tumour-intrinsic processes (non-genetic programs permissive to autocrine EGFR signalling) or stimulation from microenvironment cells (paracrine EGFR signalling) remains to be determined.

The potential utility of mutant-specific^27^^,^^28^ and pan-KRAS^29^ inhibitors (KRASi) is eagerly awaited in BTC. However, the presence of oncogenic mutations is alone insufficient for maximal therapeutic response, with functional T cells being identified as critical for the antitumour effects of KRASi in mice.^28^ Distinct immunological subgroups of iCCA,^30^ eCCA,^31^ and GBC^32^ with different biology and clinical outcomes have been reported among patients. Anticipating the concordance of mouse observations with humans, immune-mediated primary resistance to targeted therapies might be a new challenge on the horizon for a subgroup of patients with *KRAS*-mutated disease.

It is important to note that some tumours harbour alterations predisposing them to rapid adaptation (for example, *TP53* mutations^33^^,^^34^) under treatment challenge. These molecular alterations may not directly impact the mechanism of action of the targeted therapy, but instead confer more aggressive biology independent of treatment type. With standard radiological evaluation scheduled at 3-month intervals, it is likely that disease with rapidly acquired resistance is masquerading among those with true primary resistance. This is consistent with *TP53* mutations being associated with diminished targeted therapy^7^ and chemotherapy benefit^35^ in BTC, as well as poor prognosis generally.^36^^,^^37^

## Considerations and strategies to improve application of targeted therapy

**Figure 2:**
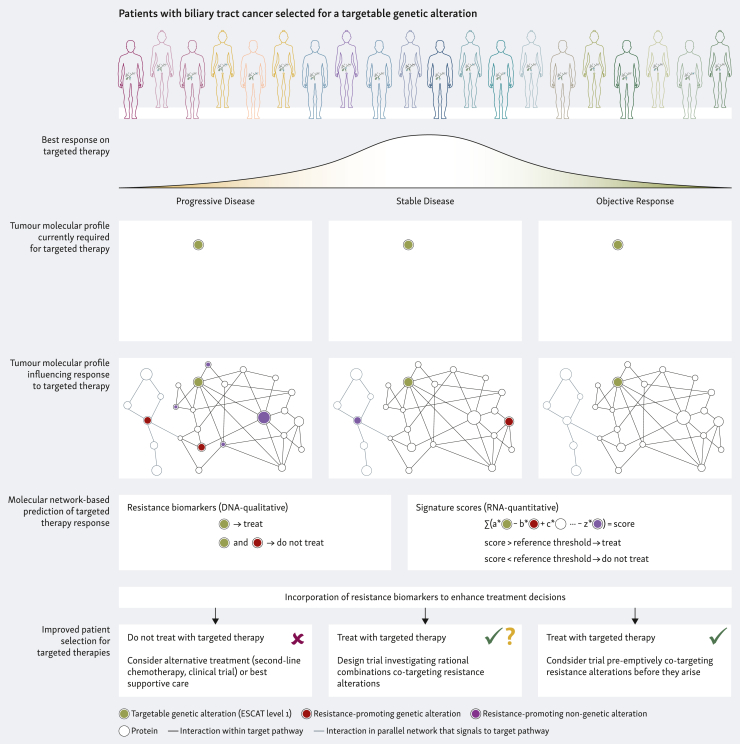
**Current criteria, limitations, and future strategies to refine molecular selection of patients with biliary tract cancer for targeted therapies.** ESCAT, ESMO Scale of Clinical Actionability for molecular Targets.

Resistance (primary and/or secondary) is an unfortunate inevitability for all patients with BTC that should be tackled up-front. Comprehensive molecular evaluation of sensitivity and resistance mechanisms operative in a patient’s tumour before treatment as well as combination regimens co-targeting (potential) resistance mechanisms are necessary next steps to improve the use of targeted therapies (Figure 2). These principles apply to all upcoming and future targeted therapies.

Given the diversity of alterations (genetic, non-genetic) and signalling networks influencing targeted therapy response, collective efforts must now be directed to refine the current molecular criteria for patient selection beyond the status of the target alteration.^38^ One strategy could simply be to determine the presence of co-alterations known to undermine therapeutic benefit, similar to how *KRAS* mutations are used as molecular contraindications for cetuximab in advanced colorectal cancer.^39^ If the negative impact of RAS/mitogen-activated protein kinase pathway activation on FGFRi sensitivity in preclinical studies^23^, ^24^, ^25^ is also found to be consistent with patient response data (clinical trial or real world), then exclusion of patients with these co-alterations from FGFRi protocols should be considered.

Alternatively, using RNA signatures to predict targeted therapy response has several advantages over qualifying the co-occurrence of single-gene DNA alterations. Specifically, targeted therapy response is influenced by many genes, only a minority of which have DNA alterations but many of which have RNA alterations (changes in expression levels due to altered pre- and post-transcriptional regulation). Further, response is also influenced by microenvironment cells which are not altered at the DNA level but are substantially altered at the RNA level. RNA is also a quantitative variable, enabling the relative expression of one gene to be compared to others (unlike genetic alterations that are typically annotated as present or absent). RNA signatures (encompassing many predictive genes in a linear formula) predictive of chemotherapy benefit have been proposed in iCCA^35^ and pancreatic cancer,^40^ as well as used to guide treatment prioritisation in breast cancer.^41^ However, developing such signatures for individual drugs in BTC will be labour-intensive, requiring: (i) retrospective comparison of RNA profiles from pre-treatment biopsies to identify an initial response signature; (ii) retrospective optimisation of the RNA signature in a training cohort, including establishment of reference values determining when a patient is predicted to benefit or not benefit from the treatment; (iii) retrospective and prospective validation of the optimised signature. This is certainly challenging for a rare cancer type in which only a minority of tumours harbour the actionable alteration of interest, underscoring the importance for molecularly analysing pre-treatment biopsies from historical landmark trials.

A devil’s advocate perspective could argue whether such extensive molecular developments should be prioritised to avoid non-beneficial targeted therapies being given to a subgroup of non-responders, given the modest duration of disease control among responders. While this may be true, molecular identification of primary and secondary resistance mechanisms is providing a blueprint for the design of rational combination therapies. Already, combining FGFRi with inhibitors of mitogen-activated protein kinase kinase-MAPKK (binimetinib, trametinib),^23^, ^24^, ^25^ alpha serine/threonine-protein kinase (TAS0612, TAS-117),^23^ ErbB (afatinib),^26^ or KRAS (deltarasin)^24^ boosts therapeutic response in preclinical models, including those exhibiting primary resistance to FGFRi monotherapy. A phase I basket trial combining pemigatinib with afatinib for FGFR-altered tumours is scheduled to begin recruiting in 2024 (NCT06302621). Moving forward, such combination therapies represent molecularly rationalised approaches to increasing the proportion of responders and extend the duration of response to targeted therapies.

## Conclusion

Supported by clinical and biological data, BTC has established itself as a model disease for precision medicine approaches. While efforts are ongoing to evaluate benefit from targeted therapies in first line (NCT03656536, NCT04093362, NCT05615818), existing bugs in precision approaches must also be resolved independent of their treatment sequencing. Refining molecular criteria for patient selection and biologically rationalised design of combination therapies are ultimately needed to ensure the right patient is provided the right regimen at the right time.
